# Management of a Dentigerous Cyst: A Two-Year Review

**DOI:** 10.5005/jp-journals-10005-1100

**Published:** 2010-04-15

**Authors:** Anjana G, Balagopal Varma, Ushus P

**Affiliations:** 1 Professor, Department of Pediatric Dentistry, Amrita School of Dentistry, Amrita Institute of Medical Sciences and Research Center, Kochi, Kerala, India; 2 Vice Principal, Professor and Head, Department of Pediatric Dentistry, Amrita School of Dentistry, Amrita Institute of Medical Sciences and Research Center, Kochi, Kerala, India; 3 Reader, Department of Oral and Maxillofacial Surgery, Amrita School of Dentistry, Amrita Institute of Medical Sciences and Research Center, Kochi, Kerala, India

**Keywords:** Dentigerous cyst, Marsupialization.

## Abstract

Dentigerous cyst is reported to be one of the most common odontogenic cysts of the jaws.^1,2^ The most commonly involved teeth are mandibular third molars, maxillary canines, maxillary third molars and rarely maxillary anteriors.^2,3^ This case report is about the management of a dentigerous cyst associated with a mandibular second molar (37) in a 9-year-old girl by a conservative treatment plan of marsupialization of the cyst.

A 2-year postsurgical review reveals complete disappearance of the cystic lesion and normal alignment of 37 and 38.

## INTRODUCTION

Dentigerous cyst is the most common type of developmental odontogenic cyst, making up about 20 to 24% of all epithelium-lined cysts of the jaws.^4-6^ It encloses the crown of an unerupted tooth and is attached to the CEJ.^5^ Dentigerous cyst arises as a result of cystic change in the remains of the enamel organ after enamel formation is complete.^1,5^ They are more than twice common in males than in females.^6^

Although most dentigerous cysts are considered to be of developmental origin, some examples seem to have an inflammatory pathogenesis, but it is impossible to determine histopathologically whether the inflammatory component is primary or secondary in nature.^5^ Accumulation of fluid occurs between the layers of reduced enamel epithelium or between enamel epithelium and enamel due to obstruction of venous outflow and inducement of serum transudation across the capillary wall, as a result of compression of tooth follicle by the erupting tooth.^1,3,7^ Dentigerous cysts can grow to a considerable size and large cysts may be associated with painless expansion of the jaw in the involved area. Extensive lesions cause facial asymmetry.^5^

## CASE REPORT

**Fig. 1:**
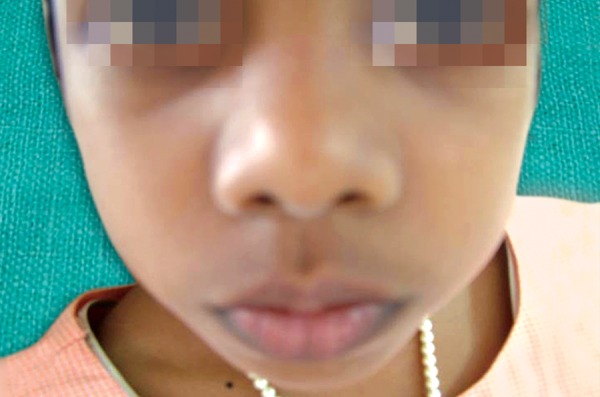
Extraoral appearance of the lesion

**Fig. 2:**
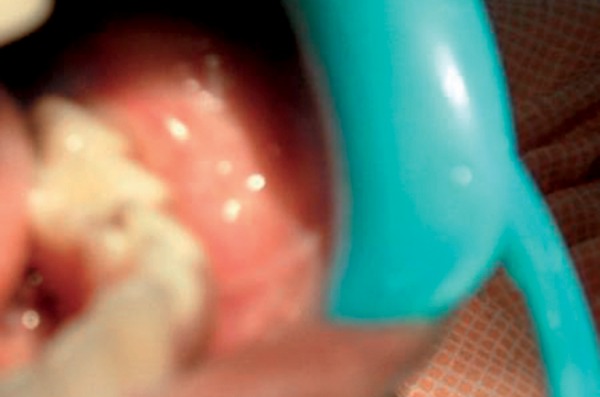
Intraoral appearance of the lesion

**Fig. 3:**
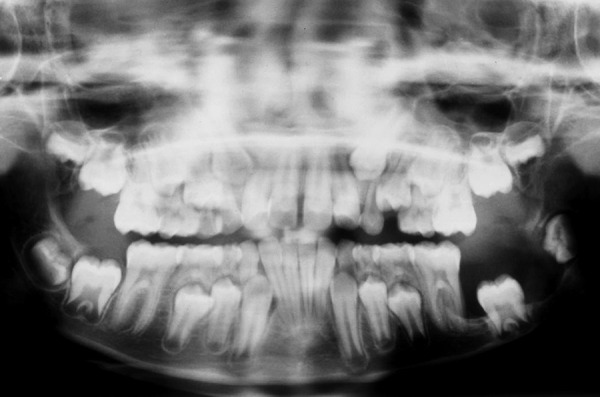
Radiographic appearance

An 8-year-old girl reported to the Department of Pediatric Dentistry, Amrita School of Dentistry, with a painful, progressive, hard swelling of left side of the face and left molar area causing facial asymmetry (Fig. 1). Clinical examination revealed a nonfluctuant swelling on the left buccal vestibule adjacent to the molar teeth (Fig. 2). Size of the lesion was 2 × 4 cm clinically. Orthopantomograph revealed a large circumscribed swelling around the crown of left mandibular second molar displacing the tooth bud of the third molar distally (Fig. 3). Result of FNAC was suggestive of a cystic lesion. This along with the clinical and radiographic appearance of the lesion led to a provisional diagnosis of dentigerous cyst.

It was decided to manage the lesion by marsupialization and an acrylic appliance to prevent the closure of the surgical opening, to attempt and salvage the involved permanent molar as a preliminary step in management with enucleation deferred until if need arises. Nowadays marsupialization of the cyst lining is the treatment of choice for dentigerous cyst in children in order to give a chance to the unerupted tooth to erupt.^7^

**Fig. 4:**
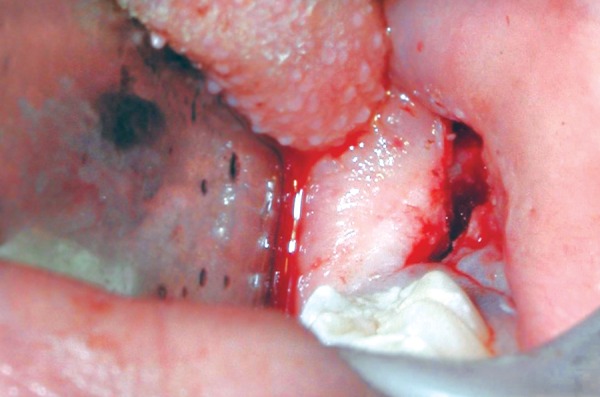
Intraoperative picture of the surgical window

**Fig. 5:**
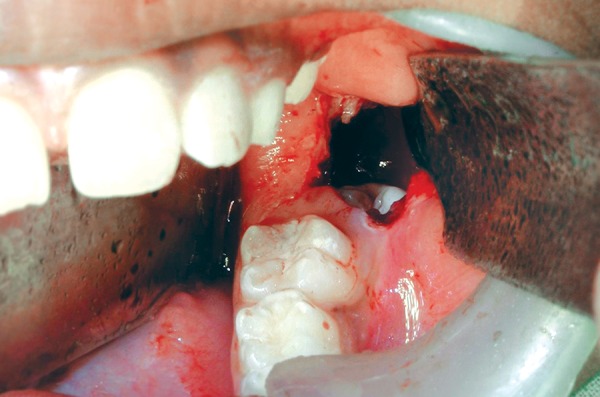
Intraoperative picture of the surgical window and 37 in the cystic space

After routine blood investigations and preoperative serological evaluations, marsupialization of the lesion was done under general anesthesia (Figs 4 and 5).

**Fig. 6:**
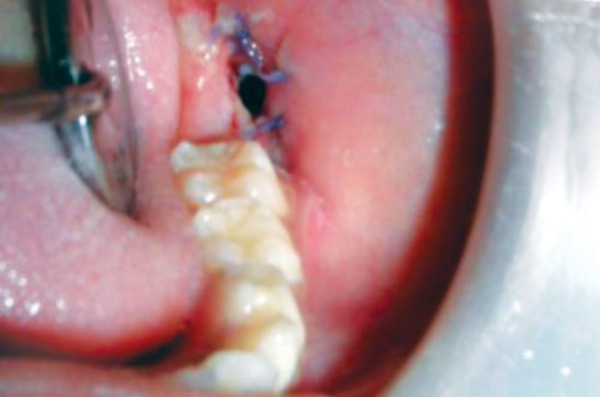
Postsurgical―10th postoperative day

**Figs 7A and B:**
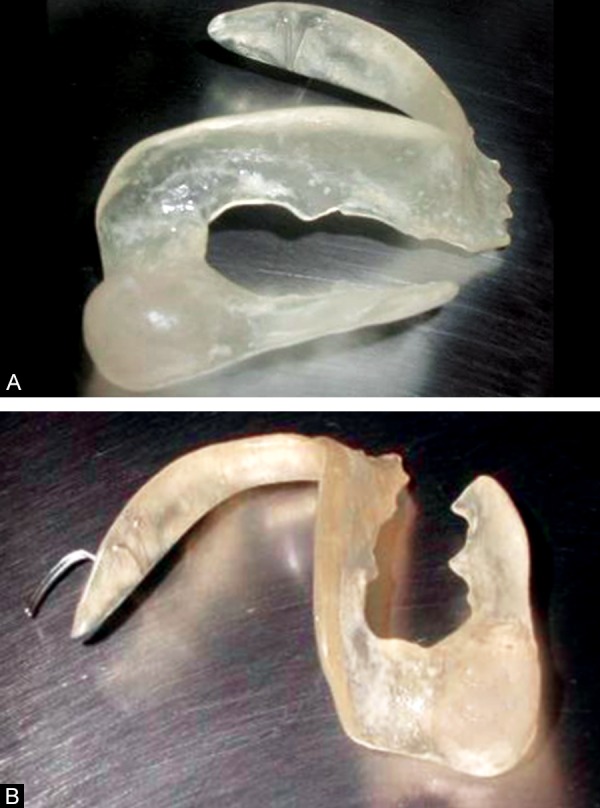
Acrylic plate

**Fig. 8:**
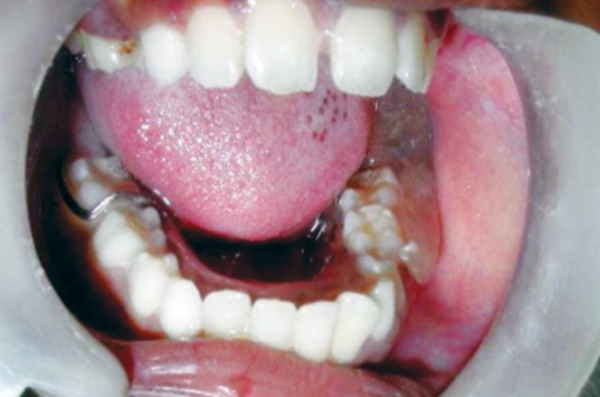
Acrylic plate in position to keep the surgical window open

**Fig. 9:**
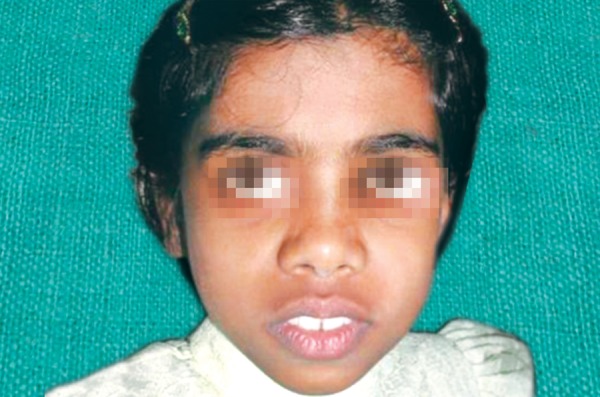
Two-month postoperative extraoral appearance. Facial asymmetry is absent

**Fig. 10:**
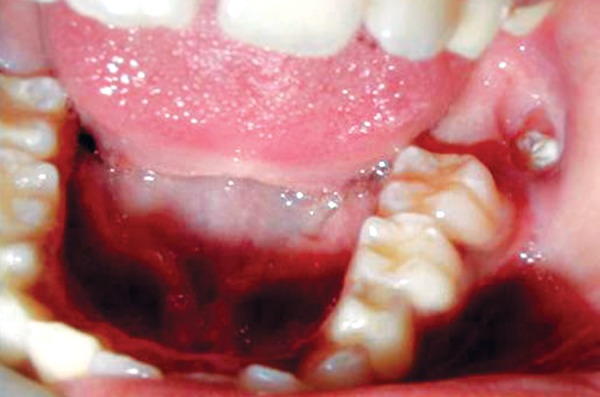
Two-month postoperative intraoral―37 erupting into the surgical opening

**Fig. 11:**
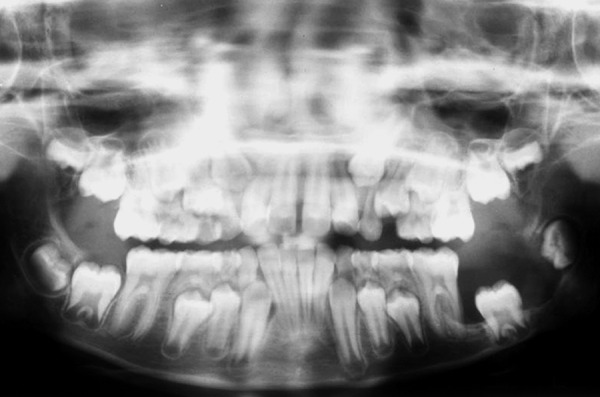
OPG―2-month postoperative lesion has reduced in size, tooth buds of 37 and 38 are moving to normal position

**Fig. 12:**
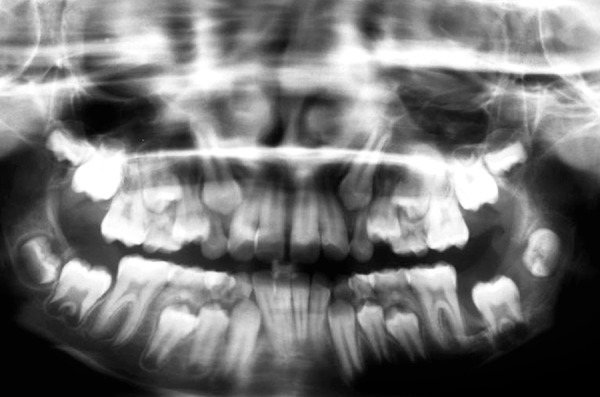
OPG―6-month postoperative size of the lesion reduced considerably and 37 shows normal and progressive root formation

**Fig. 13:**
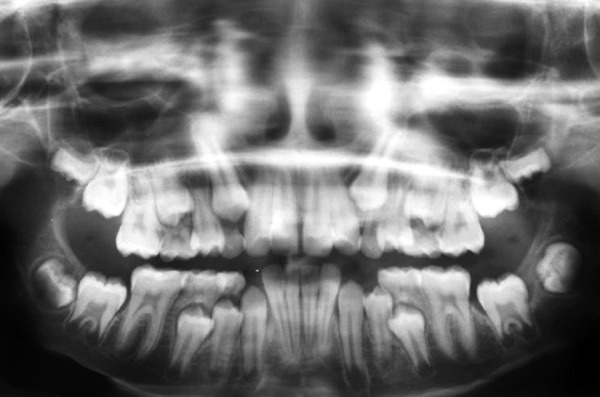
OPG―1-year postoperative complete disappearance of the lesion with 37 and 38 in normal anatomic position

**Fig. 14:**
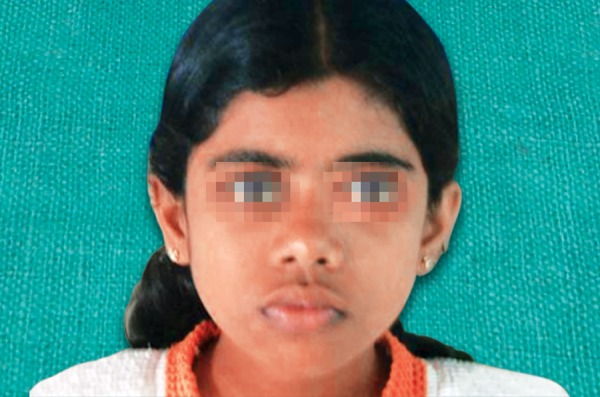
Two-year postoperative extraoral appearance

**Fig. 15:**
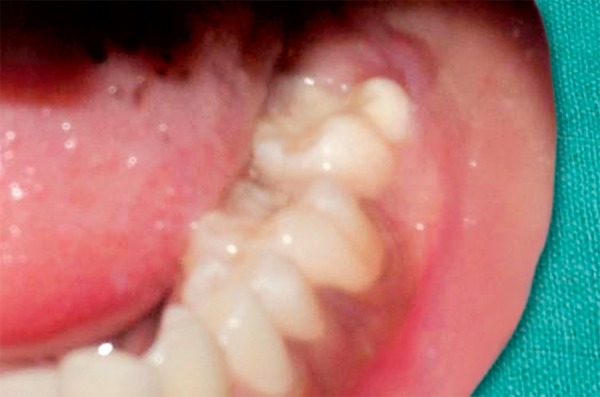
Two-year postoperative intraoral appearance

**Fig. 16:**
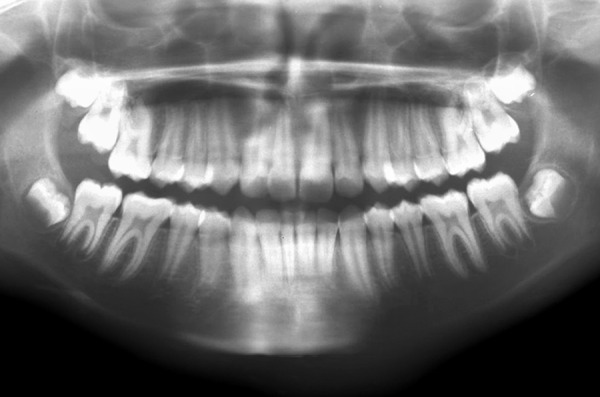
OPG―2-year postoperative permanent dentition with 37 achieving complete occlusion and root formation

Marsupialization refers to creating a surgical window in the cyst wall, evacuating the contents of the cyst, and maintaining the continuity between the cyst and the oral cavity.^8^ The cyst did not show any signs of infection, except that the patient had mild pain on presentation. The cystic contents were not purulent. The epithelial lining showed signs of inflammation. A part of the cystic lining was removed for histopathological evaluation. Impression of the surgical opening and mouth of the lesion was made. Antibiotics were prescribed to prevent secondary infection postsurgically. The regimen followed was amoxycillin 250 mg thrice daily for five days. Histopathological evaluation of the specimen showed a thick fibrocollageneous cyst wall showing sparse inflammation with an ulcerated squamous lining consistent with dentigerous cyst confirming the diagnosis. Acrylic appliance was fabricated and inserted on the tenth postoperative day to maintain the surgical window (Figs 6 to 8) open and was reviewed bimonthly. Within two months the appliance became ill fitting due to the eruption of the involved molar (Figs 9 to 11). A radiological and clinical evaluation was done for a period of two years, which revealed a progressive reduction in size, and disappearance of the lesion completely. The displaced third molar bud moved into position, and the involved molar erupted in alignment and achieved occlusion uneventfully without assistance (Figs 12 to 16).

## DISCUSSION

A cyst is defined as an epithelium lined sac filled with fluid or soft material.^8^

The prevalence of cysts in the jaws can be related to the abundant epithelium that proliferates in the bone during the process of tooth formation and along the lines where the surfaces of embryologic jaw processes fuse. Those arising from the odontogenic epithelium are odontogenic cysts and those arising from the oral epithelium trapped between fusing processes during embryogenesis are called fissural cysts.^8^

Dentigerous cysts are odontogenic cysts arising from the reduced enamel epithelium after the formation of the tooth crown. The lower-third molars and the upper canines are the most affected, but any permanent tooth can be involved.^6^ In this case, the tooth involved was a permanent second molar (37), which is not very common.

Radiographically the dentigerous cyst typically appears as a well-circumscribed, unilocular, usually symmetric radioluscency larger than that of a normal dental follicle around the crown of the tooth.^2,5^ Radioluscency often have a sclerotic border indicating bony reaction.^1,4^ An infected cyst may show ill-defined borders.^5^ The cyst to crown relationship in a dentigerous cyst can show different variations. The cyst surrounds the crown of the tooth with the crown projecting into the cyst in central variety. In the present case, the cyst appeared to be that of central variety with the crown of the tooth projecting into the cyst lumen.

When the cyst grows laterally along the root surface surrounding the crown partially it is called the lateral variety as seen with mesioangular impacted mandibular third molars. In the circumferential variety, a considerable amount of root appears to lie within the cyst along with the crown that is surrounded by the cyst.^5^

Histologically the wall of the dentigerous cyst is composed of thin connective tissue containing varying numbers of islands of odontogenic epithelium with a layer of stratified squamous epithelium lining the lumen of the cyst. Inflammatory cell infiltration of the connective tissue is common. Presence of Rushton bodies which are peculiar, often curved, hyaline bodies probably of hematogeneous origin are seen within the epithelium lining. The content of the cyst lumen is a watery yellow fluid, which can occasionally be blood tinged.^1^ During marsupialization of the cyst, it was observed that the cyst contents were watery yellow and not turbid or purulent, indicating that the cyst was not infected.

The dentigerous cyst is potentially capable of becoming an aggressive lesion. Expansion of the bone, facial asymmetry, extreme displacement of teeth, severe root resorp-tion of adjacent teeth and pain are all possible sequelae of continuous enlargement of the cyst.^1^ Except root resorption all other clinical features of an expanding dentigerous cyst were seen in this case.

Potential complications are development of ameloblasto-ma, epidermoid carcinoma or a mucoepidermoid carcinoma.^1^

Though the usual treatment for a dentigerous cyst is careful enucleation of the cyst together with the removal of unerupted tooth, if eruption of the unerupted tooth is considered feasible, the tooth may be left in place after partial removal of the cyst wall.

This permits the decompression of the cyst with a resulting reduction in the size of the bone defect. Some patients may need orthodontic treatment to assist eruption. Large dentigerous cysts may also be treated by marsupialization.^5,8^ Considering the age of the patient in this case, it was important to prevent the loss of a permanent tooth, which in turn will create the need for prosthesis. Hence, enucleation was decided against and the outcome turned out to be rewarding. The involved tooth erupted and was well aligned in the arch without any orthodontic assistance.

## CONCLUSION

Marsupialization can be thought of as the first treatment option for dentigerous cyst, in children, when loss of viable permanent tooth buds can be prevented.

Marsupialization allows for guided eruption of the developing teeth as the overlying cystic structure is decompressed.

A radiographic review in every 6 months for the first 2 years is mandatory since the remnants of cystic lining can undergo ameloblastic changes.
